# Postpartum Ovarian Vein Thrombosis: Two Cases and Review of Literature

**DOI:** 10.1155/2009/101367

**Published:** 2009-09-30

**Authors:** Amos A. Akinbiyi, Rita Nguyen, Michael Katz

**Affiliations:** ^1^Department of Obstetrics and Gynaecology, Regina General Hospital, University of Saskatchewan, Regina, Canada S4S 0A5; ^2^Department of Surgery, College of Medicine, University of Saskatchewan, Saskatoon, Canada S7N 0W8; ^3^Department of Radiology, Royal University Hospital, College of Medicine, University of Saskatchewan, Saskatoon, Canada S7N S7N

## Abstract

*Introduction*. We presented two cases of late presentation of ovarian vein thrombosis postpartum following vaginal delivery and cesarean section within a short period in our institution. Both of them had pelvic pain following their deliveries which was associated with fever and chills. One of them was quite a big-sized thrombophlebitic vein which was about 10 × 6 × 5 centimeters following a computed tomography. They were both treated initially for urinary tract infection, while a large ovarian vein thrombosis was not diagnosed in the second patient until her emergency department admission. *Conclusion*. Ovarian vein thrombosis is rare, but could present late, and difficult to diagnose, hence, should be considered as a differential diagnosis in a postpartum woman with fever and tender pelvic mass.

## 1. Introduction

Ovarian vein thrombosis is an uncommon but potentially serious disorder that is associated with a variety of pelvic conditions—most notably, recent childbirth. It could also be associated with pelvic inflammatory disease, malignancies, and pelvic surgery. Ovarian vein thrombosis occurs in 0.05–0.18% of pregnancies and is diagnosed on the right side in 80–90% of the affected postpartum patients [1–3]. Prompt diagnosis and treatment of this condition is needed to avoid the morbidity and mortality that are related both to the thrombosis and to any associated infection/sepsis. One of these cases illustrates the importance of including ovarian vein thrombosis as a differential diagnosis in women who present in the postpartum period with a tender pelvic mass.

## 2. Case Presentation

### 2.1. Case 1

A 26-year-old woman presented at 13 days postpartum to an emergency department with severe, stabbing, right flank pain. The pain had been present since postpartum day 2, with associated fever (temperature 39.5 degrees centigrade) and chills. At that time, diagnosis of urinary tract infection was made by urinalysis and culture which confirmed *Escherichia coli* as the infective organism, and the patient was treated with amoxicillin based on the sensitivity results. Unfortunately, the pain did not resolve. There was no associated vagina bleeding, nausea, or vomiting. 

On examination, the patient was afebrile and had tenderness in the umbilical and right flank area. Ultrasonography was not performed after the delivery because it was not considered appropriate.

Her antenatal period was uneventful. She had a spontaneous vaginal delivery of a live born-term female. The immediate postpartum period was unremarkable. There was no other significant past medical or surgical history. 

**Figure 1:**
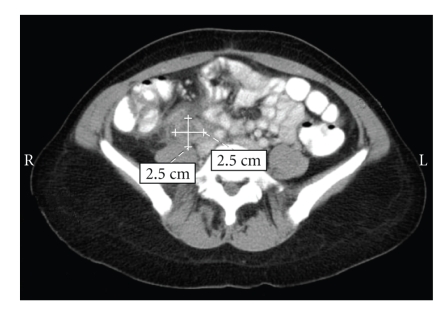
Longitudinal section right and left ovarian veins case 2.

Investigations showed white blood cell count of 12.6 × 10^9^/L, hemoglobin of 114 g/L, and reactive thrombocytosis with a platelet count of 587 × 10^9^/L. The rest of the laboratory investigations were within normal limit. A pelvic computed tomography showed findings consistent with a thrombosed right ovarian vein, measuring 8 × 5 × 5 cm (Figure 1).

A consult to internal medicine was subsequently made, the diagnosis confirmed, and the patient was initiated on therapy of low-molecular-weight heparin (Dalteparin sodium) at a dose of 12,500 units/day by subcutaneous injection and discharged home, to be followed up with internal medicine.

### 2.2. Case 2

A 19-year-old G3 P 2-0-1-3 female presented 3 weeks after a lower segment cesarean section for monochorionic diamniotic twins with a right-sided abdominal mass and abdominal pain and cramping of 1-week duration. The pain was described as gas-like, nonradiating, and admitted to passing flatus and a bowel movement that day. The patient was afebrile, and also denied nausea, vomiting, diarrhoea, and difficulty with voiding but did admit to a fever the previous week. Her fever was not based on any objective measurement. Her babies were reported to be doing well. The patient was otherwise healthy with no allergies and only taking iron. The patient was nonsmoker and denied alcohol or drug use. 

On physical examination, the patient looked well with normal vital signs. Her abdomen was distended, nontender, and an 8 cm × 10 cm mass was found below the right costal margin with a consistency of an ovarian mass. The mass felt irregular in consistency. Her incision had healed well. On pelvic examination, a 10-centimeter long mass was felt in the right lower abdominal region which was slightly mobile and nontender, which extends from just below the right renal vein down to the right iliac fossa. The uterus was barely palpable above the pubic symphysis which was considered normal.

**Figure 2:**
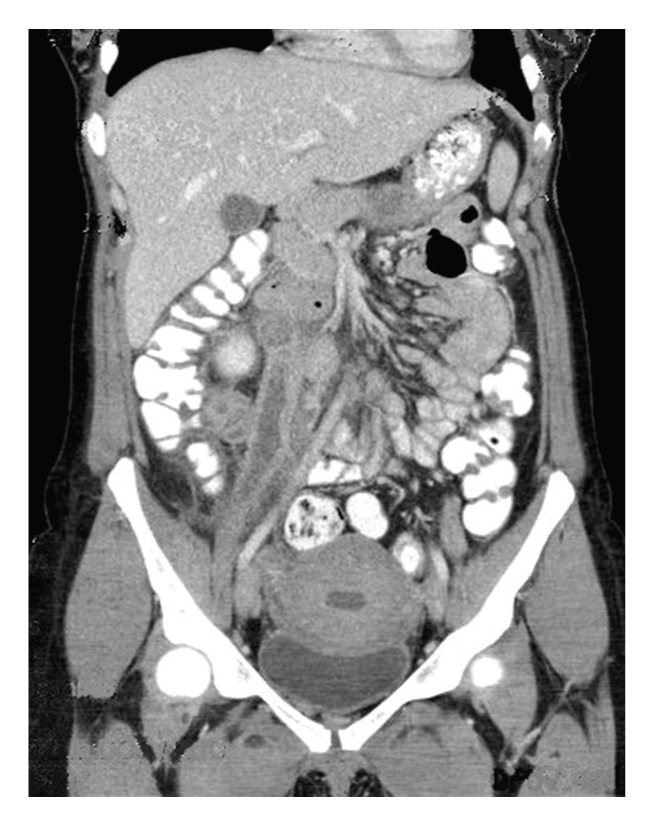
Coronal section right ovarian vein case 1.

**Figure 3:**
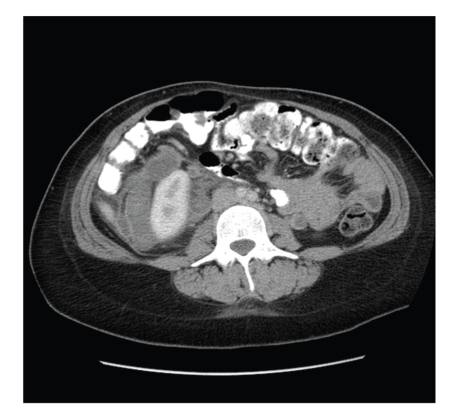
Coronal section right ovarian vein case 2.

The rest of her physical examination was unremarkable. Complete blood count and an abdominal ultrasound showed numerous hypoechoic tubular structures just inferior to the right kidney. A computed tomogram of the abdomen/pelvis with contrast identified numerous nonenhancing dilated tubular structures extending from the right renal vein down to the ovary measuring 10 × 6 × 5 cm. The left side also showed a similar but less obvious structure (see Figures 2 and 3). There was also found a large amount of air within the endometrial cavity concerning for endometritis. The patient was admitted and treated as a pelvic septic thrombophlebitis and anticoagulated. She was commenced on low-molecular weight heparin at a dose of 12,500 units per day while Cefazolin 1 gm was given every 8 hours intravenously for five days. The patient had an uneventful stay in hospital and was discharged home after 5 days with a followup-computed tomogram in two weeks. She was also given an appointment to see her primary care provider in one week if any problem arise. 

## 3. Discussion

Women are five times more likely to suffer from a thromboembolic event when they are pregnant1. The overall incidence of thromboembolic events ranges from 0.3% to 1.2% [2]. The most common postpartum thromboembolic events include deep vein thrombosis and pulmonary emboli. However, ovarian vein thrombosis complicates 0.05%–0.18% of pregnancies [3–5]. 

The first case of postpartum ovarian vein thrombosis was described by Austin in 1956 [6]. The pathophysiology of ovarian vein thrombosis is ascribed to Virchow's triad of hypercoagulability, venous stasis, and endothelial trauma. Pregnancy is a period where women are at a hypercoagulable state due to normal physiological changes. These changes include an increase in clotting factors such as factors VII, VIII, IX, X, XII, vWF, and fibrinogen. As well, free levels of protein S are decreased. There is venous stasis of the lower limbs due to compression of the pelvic veins and inferior vena cava by the uterus. Increased levels of estrogen and increased local production of nitric oxide and prostacyclin also contribute to increased deep vein capacitance. 

Endothelial trauma can occur at the time delivery or from local inflammation. These pregnancy-induced changes help protect women from hemorrhagic complications during placentation and labour; however, they also place women at an increased risk of venous thromboembolic diseases. 

The right ovarian vein is implicated in 90% of cases of ovarian vein thrombosis [3]. Several explanations have been proposed for this skew towards the right ovarian vein ranging from retrograde drainage from the left ovarian vein and anterograde flow into the right ovarian vein in the postpartum setting to dextrorotation of the enlarging uterus, causing compression of the right ovarian vein and right ureter as they cross the pelvic brim and the fact that the right ovarian vein is longer than the left, and when dilated, the valves become incompetent, making it easier for a thrombus to form [2, 3, 7, 8]. 

Patients with ovarian vein thrombosis typically present with fever, pelvic pain, and a “ropelike” palpable abdominal mass [5, 9]. Case 2, however, did not present with fever, but she gave a history of being feverish few days prior to her second admission. We, however, decided to treat her with antibiotics despite her lack of any evidence of fever. We do not understand the reason for air in her uterus but we were very suspicious of endometritis hence we considered to treat her with antibiotics. Case 1, however, was discharged prematurely with the followup to be conducted by her family physician. This patient should have been kept for few more days in hindsight. The incidence peaks around postpartum day 2 for full-term deliveries and occurs within 10 days postpartum in 90% of cases [9]. As symptoms are nonspecific, the diagnosis of ovarian vein thrombosis may be delayed. The differential diagnosis for ovarian vein thrombosis includes appendicitis, endometritis, adnexal torsion, pyelonephritis, and septic pelvic thrombophlebitis. Ovarian vein thrombosis is differentiated clinically from septic thrombophlebitis in that patients with septic thrombophlebitis appear clinically well but have continuing high spiking fevers, but the physical examination is also normal [3]. 

The diagnosis of ovarian vein thrombosis is ideally made with pelvic CT scanning, which will show an enlarged vein with a low-density lumen and sharply defined walls [9, 10]. However, ultrasound is commonly used as the first radiographic investigation in postpartum women. Ultrasound scan in Case 2 was not conclusive, but the computed tomogram enabled us to make a definitive diagnosis. Ovarian vein thrombosis on ultrasound will appear as an anechoic to hypoechoic mass between the adnexa and the inferior vena cava, with absence of blood flow [3]. The sensitivity of CT scanning for diagnosing ovarian vein thrombosis is 100%, and 52% for Doppler ultrasonography [2]. Magnetic resonance image is considered ideal for its sensitivity and lack of ionizing radiation. 

Treatment for ovarian vein thrombosis includes antibiotics and anticoagulation. Appropriate antibiotics include clindamycin, or gentamicin, or a second- or third-generation cephalosporin. Although low-molecular-weight heparins have been shown to be as effective as unfractionated heparin for treating ovarian vein thrombosis, the studies providing this evidence are of small design with unsatisfactory data. Further investigation is required to determine if low-molecular-weight heparins are appropriate to use in treatment of ovarian vein thrombosis [3, 9]. We were quite concerned about the size of the thrombosed ovarian vein in Case 2 despite that, without any evidence of fever, she was given Cefazolin to which she responded quite well.

Complications of ovarian vein thrombosis include sepsis, thrombus extending to the inferior vena cava and renal veins, and pulmonary embolism. The incidence of pulmonary embolism is reported to be 13.2% [5]. These complications can be managed surgically with thrombectomy or with an inferior vena cava filter. Mortality due to ovarian vein thrombosis is less than 5%, most cases of which are due to pulmonary embolism [3]. Some degree of morbidity could be encountered in cases inappropriately and promptly managed.

## 4. Conclusion

Ovarian vein thrombosis, rare as it, could present late in postpartum women with serious consequences, hence a high index of suspicion for diagnosis and management is required to avoid an associated mortality and morbidity. There was no mortality in these two patients, but morbidity was reduced with prompt diagnosis and appropriate treatment.
